# Enhancement of Hard Magnetic Properties in Fraktal-Like Nano and Mesoscopic Grains

**DOI:** 10.3390/ma14061443

**Published:** 2021-03-16

**Authors:** Grzegorz Ziółkowski, Dariusz Chrobak, Grażyna Chełkowska, Ondrej Zivotsky, Artur Chrobak

**Affiliations:** 1 Institute of Physics, University of Silesia in Katowice, 75 Pułku Piechoty 1A, 41-500 Chorzów, Poland; grazyna.chelkowska@us.edu.pl (G.C.); artur.chrobak@us.edu.pl (A.C.); 2 Institute of Materials Engineering, University of Silesia in Katowice, 75 Pułku Piechoty 1A, 41-500 Chorzów, Poland; dariusz.chrobak@us.edu.pl; 3 Department of Physics, VŠB-Technical University of Ostrava, 17 listopadu 15/2172, 708 33 Ostrava-Poruba, Czech Republic; ondrej.zivotsky@vsb.cz

**Keywords:** Monte Carlo simulations, magnetic simulations, hard magnetic materials

## Abstract

The paper refers to Monte Carlo magnetic simulations for fractal-like nano and mesoscopic grains. The analyzed objects differed in the size, surface development, magnetic anisotropy and the spin values attributed to the system nodes inside the fractal. Such an approach allowed us to determine their magnetization processes as well as optimization characteristics in the direction to enhancement of hard magnetic properties. As it was shown, the size effects depend on the chosen value of magnetic anisotropy. In the case of fractals with ultra-high coercivity, the decreasing of their size leads to deterioration of coercivity, especially for the high surface to volume ratio. Opposite effects were observed for soft magnetic fractals when the nanostructure caused an appearance of the coercive field, and the maximum of energy product was predictably significantly higher than for conventional rare earths’ free permanent magnets.

## 1. Introduction

Magnetic materials are very important in modern technologies such as electromobility, magnetic cooling, or energy harvesting. Especially interesting are magnets with extreme soft or hard magnetic properties. It is well known that iron-based nanocrystalline alloys exhibit excellent soft magnetic properties (i.e., close to zero coercivity and high magnetic saturation) [1,2] that are widely applicable in electric motors and transformers. On the other side of the magnetic materials application spectra, there are hard magnets characterized by high value of the so-called energy product [3,4,5,6,7,8]. In this area, the Fe-RE-B (RE (rare earths), typically Nd, Nd/Dy, Nd/Tb) type of alloys are considered the best, showing the energy product in order of 400 kJ/m^3^. It is interesting that the classical RE-free permanent magnets reveal values of this parameter no higher than 30 kJ/m^3^ (for example—ALNICO alloys [9,10]). For economic reasons, many scientific teams have worked on searching for new RE-free/reduced hard magnetic materials that can fill the gap between these two kinds of magnets [11]. One promising research direction is nanotechnology, allowing us to obtain nanosized magnetic systems and enabling the introduction of an additional magnetic anisotropy by the interaction between nano-objects [12,13,14,15]. Surface and size effects are also important. In this context, objects with fractal-like geometry seem to be very interesting [16,17]. In some cases, procedure of fractal reproduction can be considered as a model for early-stage crystal growth, allowing generating objects with a specific surface shape. Recently, we undertook a preliminary study into the magnetization processes of such objects, i.e., 3D nanosized fractals [18]. It turned out that it is possible to control magnetic characteristics by a specific combination of magnetic properties in volume, on the surface, and by the shape of the fractal objects. The results presented in this work motivated us to significantly broaden the range of analysis, including the mesoscopic size of the objects and optimization of the energy product, that is desired for permanent magnets applications.

In this paper we have focused on simulations of magnetization processes of nano and mososcopic objects characterized by different shape and size, and different magnetic properties of the objects in volume and surface. In particular, we proposed the analysis of fractal-like objects with different ratios between magnetic moments placed in the volume and on the surface. Such objects can be considered as an equivalent of crystal grains formed during the early stages of crystallization, for example, from amorphous or liquid state. We have analyzed the magnetic characteristics of the fractals that are described by magnetically soft, hard (coercivity about 1T), and ultrahigh-coercive volume (coercivity about 8T, experimentally observed and reported in [19]) as well as different values of surface magnetic anisotropy. Such an approach allowed us to determine magnetic hysteresis loops for the studied systems, and additionally, the approach made it possible to suggest a way for designing relatively good hard magnetic materials towards reduction or elimination of RE elements in their content.

## 2. Simulation Procedure

Generally, we used the Monte Carlo Metropolis method as the simulation procedure [20]. Recently, we developed a computational approach which allows the analysis of magnetization processes of hard magnetic systems containing nano and mesoscopic 3D objects [21,22]. We introduced the following two elements: (i) modification of the cluster Wolff algorithm [23] by an additional factor taking into account information (or configuration) entropy originating from a difference in magnetic properties of system nodes [21] and (ii) formulation of scaling rules for micromagnetic simulation of mesoscopic objects [22].

**Figure 1:**
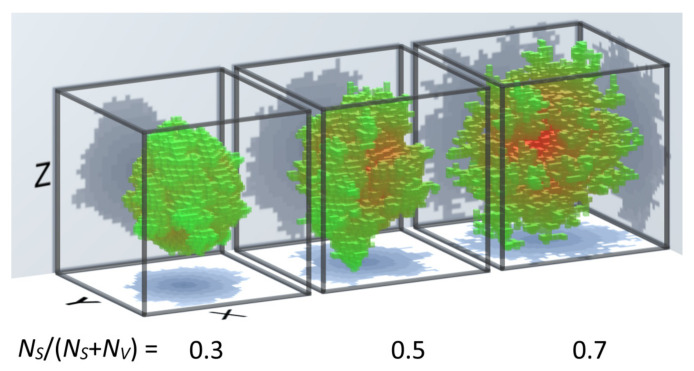
Geometry of systems obtained based on the diffusion limited aggregation (DLA) algorithm with different ratio of the number of particles on the surface (*N_S_*) to the volume (*N_V_*).

All simulated systems in this work were prepared based on the diffusion limited aggregation algorithm (DLA), described in detail in [24,25,26,27,28]. Basically, in this method, a single particle (seed) has been placed in the center of the system, and then the new particles are introduced into the system starting from the edges. The new particle moves randomly and, with some probability, it may stick to another stationary particle encountered in the system. Moreover, the algorithm was extended by a growing procedure (existing particles may extend to adjacent fields) similar to the grain growing procedure observed in the real systems. For example, the DLA can simulate chemical deposition, MBE deposition or rapid solidification from liquid state [29,30,31,32]. By controlling the attachment probability and the growth speed, it is possible to obtain the systems with a constant number of particles (7000), but with a different degree of the surface development, i.e., the ratio of the number of particles on the surface (*N_S_*) to the volume (*N_V_*), as was shown in Figure 1.

The generated fractals were embedded in 3D space divided by 40 × 40 × 40 nodes, and digitization of the system lies in assigning magnetic properties to each node. In particular, the nodes are described by a “spin vector” *S*, the definition of which is represented by the node magnetic moment, and, therefore, it can have any value and any direction. The size of the fractals were defined by a spacial distance between the nodes for the initial systems and the scaling factors. Based on the three presented systems, i.e., *N_S_*/(*N_S_* + *N_V_*) equal to 0.3, 0.5 and 0.7 (further called surface ratio), a series of simulation systems with different anisotropy values of the nodes on the surface, *K_S_* = 0 eV, 5 × 10^−4^ eV, 5 × 10^−3^ eV (with easy magnetization axes perpendicular to the surface), as well as in the volume *K_V_* = 0 eV, 5 × 10^−5^ eV and 5 × 10^−4^ eV (with easy magnetization axes parallel to the *z-axis*) were prepared. The initial distance between the nodes were 0.28 nm in order to simulate material with similar to α-Fe density. Moreover, the value of the spin varied from 0.1 to 1, and the scaling factor *n* (see the next section) varied from 1 to 100, which allows us to analyze nano and meso-sclaled objects.

The main simulation procedure consists of a series of the following repeated steps:

1. Select a random *i-*th node for further analysis and choose a random number *r*_1_ ∊ [0,1).
2. If *r*_1_ < *P_cl_* (i.e., cluster analysis probability), find a cluster around selected node and change cluster directions by *θ* angle, otherwise only the direction of the selected node is changed. In order to simulate multiphase ferromagnetic systems, it is necessary to use the disorder-based cluster MC method (which we described in [21]) with the adding probability equal to:
  (1) $$P_{ij}^{add}=\left[1-exp\left(\frac{-E_{ij}^{coupling}}{k_{B}T}\right)\right]exp\left(-\alpha S_{i}^{loc}\right)$$
  where $E_{ij}^{coupling}$ is the direct exchange coupling energy between the spins attributed to nodes *i* and *j*, $S_{i}^{loc}$ is the local configuration entropy of anisotropy (calculated in the defined sphere around the *i*-th node) and *α* is the factor responsible for strengthening and weakening of the entropy impact on the adding probability (can be randomly selected from a selected range).
3. Calculate the difference of the system energy Δ*E* before and after the change in step 2. The energy is calculated based on the 3D Heisenberg model:
  (2) $$\begin{array}{c} E=-\underset{i,j}{\sum }J_{ij}S_{i}\cdot S_{j}-\underset{i}{\sum }K_{i}{\left({\hat{s}}_{i}\cdot {\hat{n}}_{i}\right)}^{2}-g\mu_{B}\mu_{0}\underset{i}{\sum }H_{i}\cdot S_{i}\\ +D\underset{i,j}{\sum }\frac{S_{i}\cdot S_{j}-3\left(S_{i}\cdot e_{ij}\right)\left(S_{j}\cdot e_{ij}\right)}{r_{ij}^{3}} \end{array}$$
  where *J_ij_* is the exchange parameter, *S_i_* is the spin vector on site *i*, *K_i_* is the anisotropy constant (per site), ${\hat{n}}_{i}$ is the versor of the easy magnetization axis, ${\hat{s}}_{i}$ is the versor of the spin, *g* is the Lande factor, *µ_B_* is the Bohr magneton, *µ_0_* is the vacuum permeability, *H_i_* is the magnetic field on site *i*, *D* is the dipolar constant, *e_ij_* is the directional versor between the *i*-th and *j*-th nodes and *r_ij_* is the distance between them.
4. Choose a random number *r*_2_ ∊ [0,1) and if *r*_2_ > $exp\left(\frac{-∆E}{k_{B}T}\right)$ restore all changes made in step 2, otherwise accept the new configuration.

It is worth noting that the basic energy equation presented in Equation (2) is extended by the scaling rules (described in details in [22]), in order to model a large-scale mesoscopic systems, with an acceptable level of computing resource consumption. The main idea lies in the assumption that one node in the rescaled system can represent a finite volume consisting of *n* × *n* × *n* original (unscaled) nodes. In this situation, the parameters appearing in Equation (2) should be recalculated using the formulation of scaling rules in [22]. Table 1 summarizes the main simulation parameters and the scaling rules depending on the scaling factor *n*.

## 3. Results and Discussion

**Figure 2:**
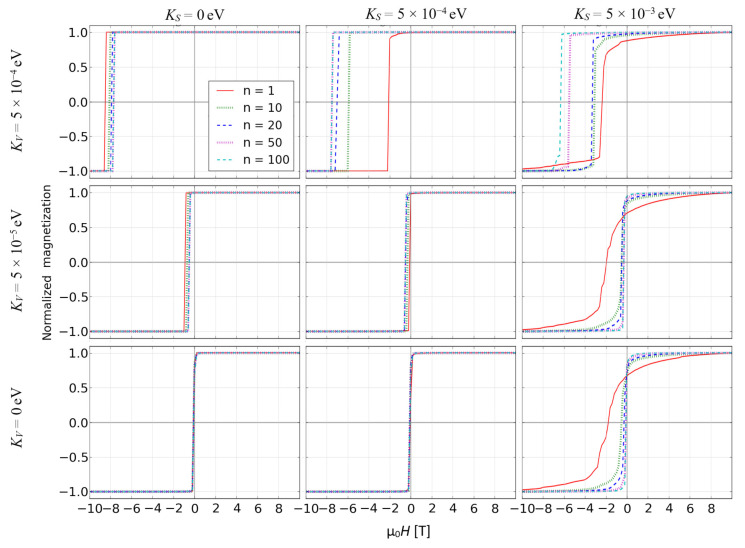
Reverse magnetization curves for all examined objects with *N_S_*/(*N_S_* + *N_V_*) = 0.7 and *S* = 1.

Magnetization processes of the objects in question were studied by carrying out a set of simulations resulting in the so-called reverse magnetization curves, i.e., gradual remagnetization of the object in opposite direction of external magnetic field after full magnetic saturation. The reverse magnetization curves were simulated for all the examined objects. Simultaneously, spin configuration was recorded in order to show coercivity mechanisms responsible for the remagnetization process. Figure 2 depicts such curves for the surface ratio equal to 0.7, *S* = 1 and for full ranges of the other simulation parameters.

**Figure 3:**
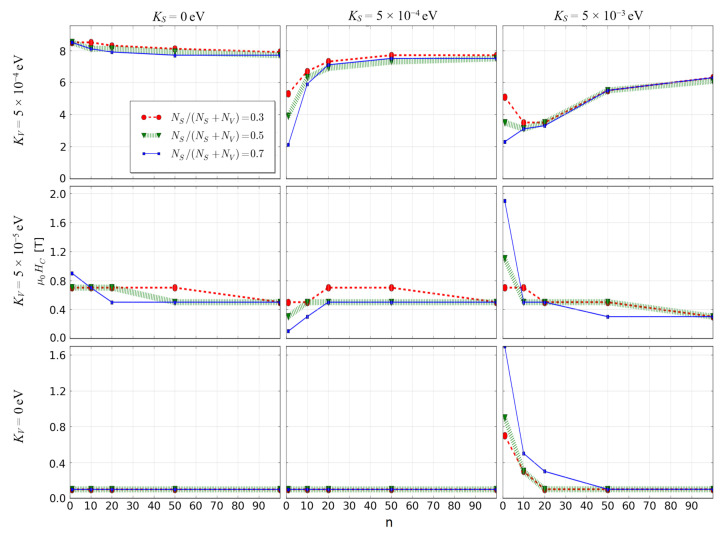
Coercive field *H_C_* as a function of the scaling factor for all studied cases with *S* = 1.

One can see that in some cases the reverse magnetization process occurs in a rapid jump, which is obviously caused by a coherent rotation of the whole spins in the fractal. In contrast to this, when the surface anisotropy is high, a graduate change of magnetization was observed. The influence of the anisotropies and scaling factor on the coercive field *H_C_* is interesting. For the fractals with ultra-high volume anisotropy, the size enlargement leads to increase of *H_C_* while for the other cases, this effect is not very strong and even opposite. For deeper analysis it is worth presenting dependencies of the scaling factor on *H_C_* for all studied cases, as shown in Figure 3. In the case of the soft magnetic fractals (i.e., *K_V_* = 0), the appearance of relatively high coercivity requires a simultaneous occurrence of their low nanometric size and high value of surface magnetic anisotropy.

**Figure 4:**
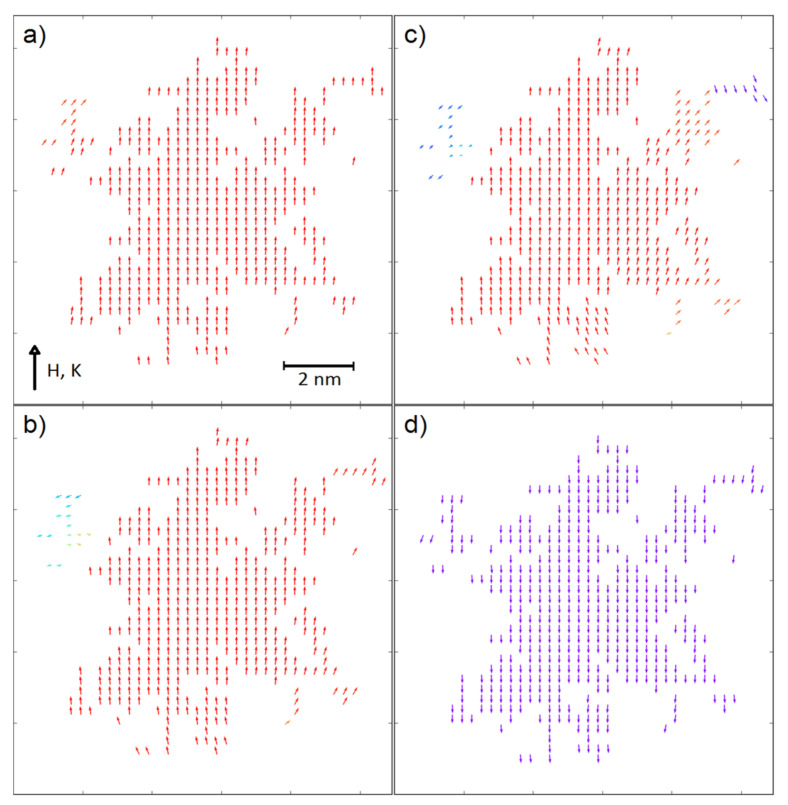
Spin configurations (cross-section) of the system with *K_S_* = 5 × 10^−4^ eV, *K_V_* = 5 × 10^−4^ eV, *n* = 1, *S* = 1 and *N_S_*/(*N_S_* + *N_V_*) = 0.7 in the external magnetic field equal to (**a**) 0 T, (**b**) −1.6 T, (**c**) −2 T and (**d**) −2.2 T.

**Figure 5:**
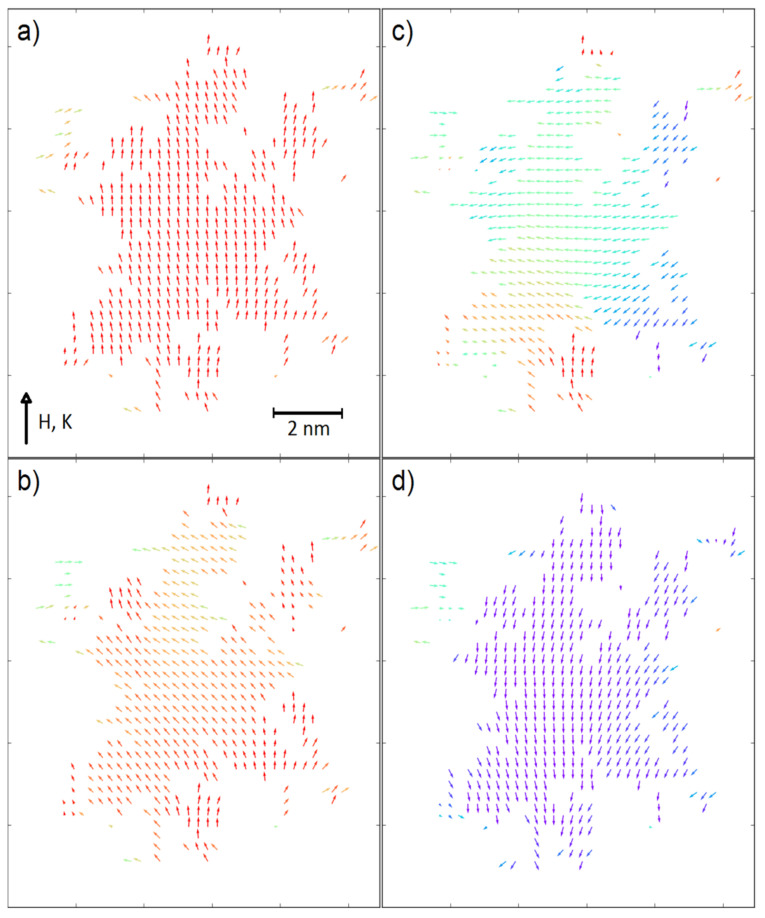
Spin configurations (cross-section) of the system with *K_S_* = 5 × 10^−3^ eV, *K_V_* = 0 eV, *n* = 1, *S* = 1 and *N_S_*/(*N_S_* + *N_V_*) = 0.7 in the external magnetic field equal to (**a**) 6.2 T, (**b**) 0 T, (**c**) −1.6 T and (**d**) −3.6 T.

In a contrast to this, for the fractals with ultra-high coercivity (i.e., *K_V_* = 5 × 10^−4^ eV and *K_S_* = 5 × 10^−4^ eV) the increasing scaling factor results in the significant increase of coercive field, especially for the surface ratio equal to 0.7. An explanation of this behavior can be found in the recorded spin configurations. Figure 4 and Figure 5 depict magnetic moments of the nodes placed on a central plane (*K_V_* = 5 × 10^−4^ eV, *K_S_* = 5 × 10^−4^ eV and *K_V_* = 0, *K_S_* = 5 × 10^−3^ eV, respectively) of the analyzed 3D fractals for the surface ratio 0.7 and with the applied external magnetic fields around the coercive field. It should be emphasized that the surface anisotropy can play different roles dependently on the *K_V_* values. When the volume anisotropy is high, the magnetic moments of the developed surface can change their directions in lower external magnetic field due to different angles between the *K_S_* vector and the field *H*; e.g., some of the surface magnetic moments are nearly parallel to the field. If magnetic coupling between volume and surface is strong enough, the anisotropy field of the whole fractal decreases. Therefore, the reverse magnetization process is initialized on surface, as shown in Figure 4. Furthermore, enlargements of the fractals to mesoscopic size is attributed to a relative decrease of the *N_S_*/*N_V_* ratio, which obviously leads to reducing the meaning of the surface magnetic moments and the increase in *H_C_* is observed (see Figure 3, middle-top graph). Relatively high surface anisotropy can cause the completely opposite effect when the volume of the fractal is magnetically soft (see Figure 3, bottom-right graph). In this situation, only magnetic moments on the surface are a source of anisotropy and via exchange interactions they can influence magnetic moments in the volume. Indeed, Figure 5 depicts such a case in which the reverse magnetization process is initiated by the soft magnetic nodes in the volume of the fractal. The effect of interaction between the volume and the surface is clearly visible in Figure 5d when the spins are aligned not perfectly co-linear, as was observed for ultra-high coercive objects (see Figure 4d).

The current part of our study can be summarized as follows. Unexpectedly, enhancement of hard magnetic properties for ultra-high coercive fractal objects is impossible in the application of higher development of the surface. It is clear that real nanosized magnets cannot occur without significant contribution of magnetic surface anisotropy. Therefore, such materials should be designed as mesoscopic grains, accounting for the fact that high contribution of the surface caused deterioration of their hard magnetic properties. For the remaining kind of fractals, i.e., with magnetic hard and soft properties, a decrease in their size together with high surface anisotropy can cause a significant increase in the coercive field.

**Figure 6:**
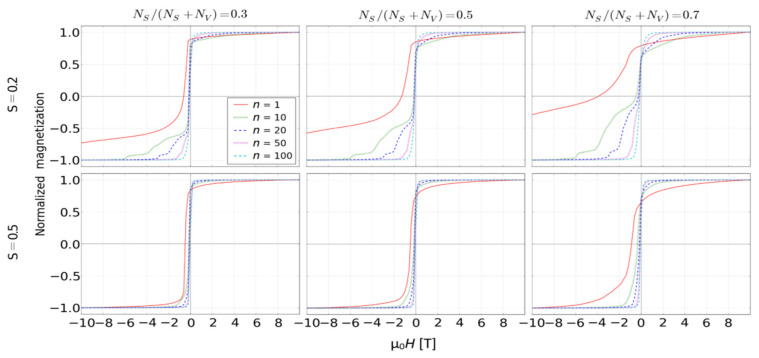
Simulated reverse magnetization curves for the systems with *K_S_* = 5 × 10^−4^ eV, *K_V_* = 0 eV and *S* = 0.2 as well as *S* = 0.5.

**Figure 7:**
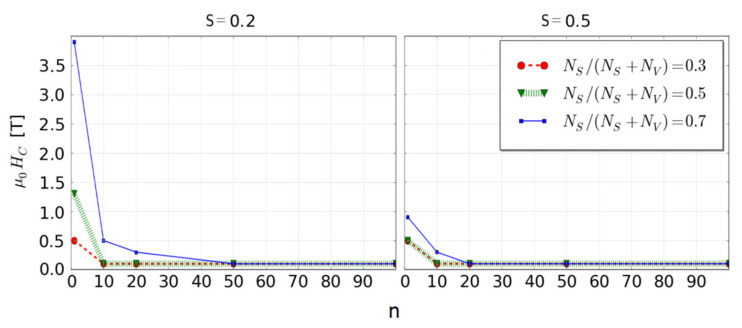
Coercive field *H_C_* as a function of the scaling factor for the systems presented in Figure 6.

The indicated possibility to create hard magnetic properties in soft magnetic fractal is extremely intriguing and worthy of further investigations. It is not a new idea; e.g., the well-known ALNICO alloys contain nanosized iron grains in the form of elongated cigars. Reviewing the literature, one can find a value of iron surface anisotropy in the order of 10^−4^ eV [33,34], so the use in our simulation of *K_S_* = 5 × 10^−4^ eV is a realistic value. Unfortunately, for *S* = 1 we did not observe any occurrence of coercivity in analyzed fractals. The reason for this could be to high magneto-static energy of the volume in a comparison with the surface anisotropy energy. The only way to obtain the desired enhancement of hard magnetic properties effect is to decrease the *S* values. For this purpose, we chose the fractal with the surface ratio equals 0.7, *K_S_* = 5 × 10^−4^ eV, *K_V_* = 0 eV and *S* ranging from 0.1 to 1. For example (i.e., for *S* = 0.2 and 0.5), Figure 6 and Figure 7 show the simulated reverse magnetization curves and the resulting *H_C_* as a function of the scaling factor, respectively. As it was expected, the decrease of the fractal magnestostatic energy (the third term in Equation (2)) caused the complexity of the reverse magnetization process that, in the case of *n* = 1, leads to an appearance of coercivity.

**Figure 8:**
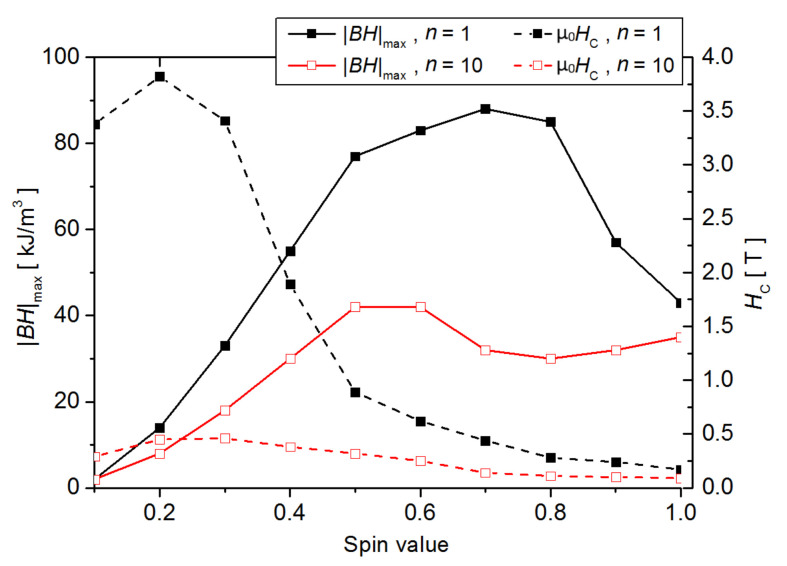
Dependencies of the *S* value on the coercive field and the estimated energy product for the systems with *K_S_* = 5 × 10^−4^ eV, *K_V_* = 0 eV, *N_S_*/(*N_S_* + *N_V_*) = 0.7 and *n* = 1 as well as *n* = 10.

Analyzing the full variability of *S,* one can notice an increase of coercive field with decreasing *S* value. Possible applications as permanent magnets also require high values of the magnetic remanence and, consequently, the energy product |*BH*|_max_. Let us estimate the |*BH*|_max_ values, taking density and magnetic induction as for *α*-Fe (i.e., induction in saturation equal to 2 T for *S* = 1). With this assumption, it is possible to plot dependencies of the *S* value on the coercive field and the estimated energy product, as shown in Figure 8 for *n* = 1 and *n* = 10.

For both quantities, the presented curves reveal maxima at different spin values. In particular, for *S* = 0.7 and *n* = 1 the energy product |*BH*|_max_ equals 85 kJ/m^3^, which is very promising considering that, for the best rare earths free permanent magnets (the ALNICO alloys), this parameter is about 30 kJ/m^3^. This means that a specific nanostructure with highly developed surface may fill the gap between conventional and rare earths-based permanent magnets. The open question is how to obtain such a structure and how to control magnetic moment of atoms in iron-based magnetic compounds. One can imagine applying rapid crystallization (flash annealing, mold suction casting, melt-spinning techniques, etc.) to control the microstructure as well as alloying addition (for example, some metaloids) to control magnetic moments attributed to Fe atoms.

**Figure 9:**
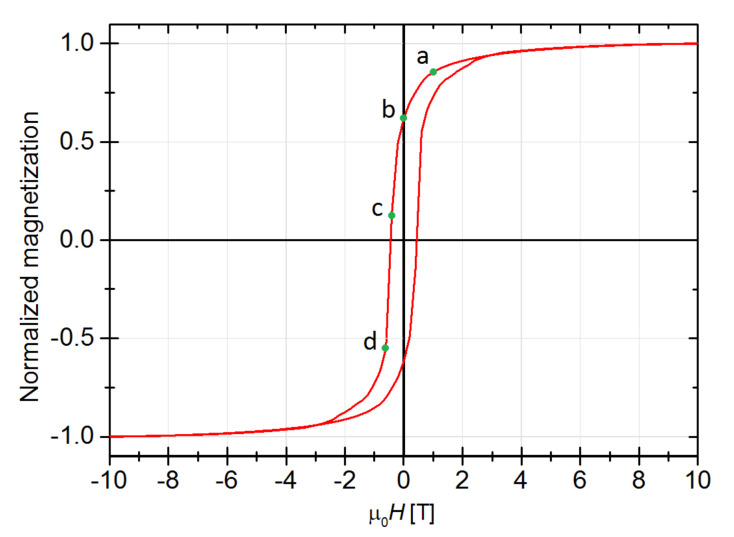
Simulated hysteresis loop for the fractal with the highest |*BH*|_max_ i.e., *S* = 0.7, *n* = 1, *K_S_* = 5 × 10^−4^ eV, *K_V_* = 0 eV and *N_S_*/(*N_S_* + *N_V_*) = 0.7. The a–d points correspond to the spin configurations in Figure 10.

**Figure 10:**
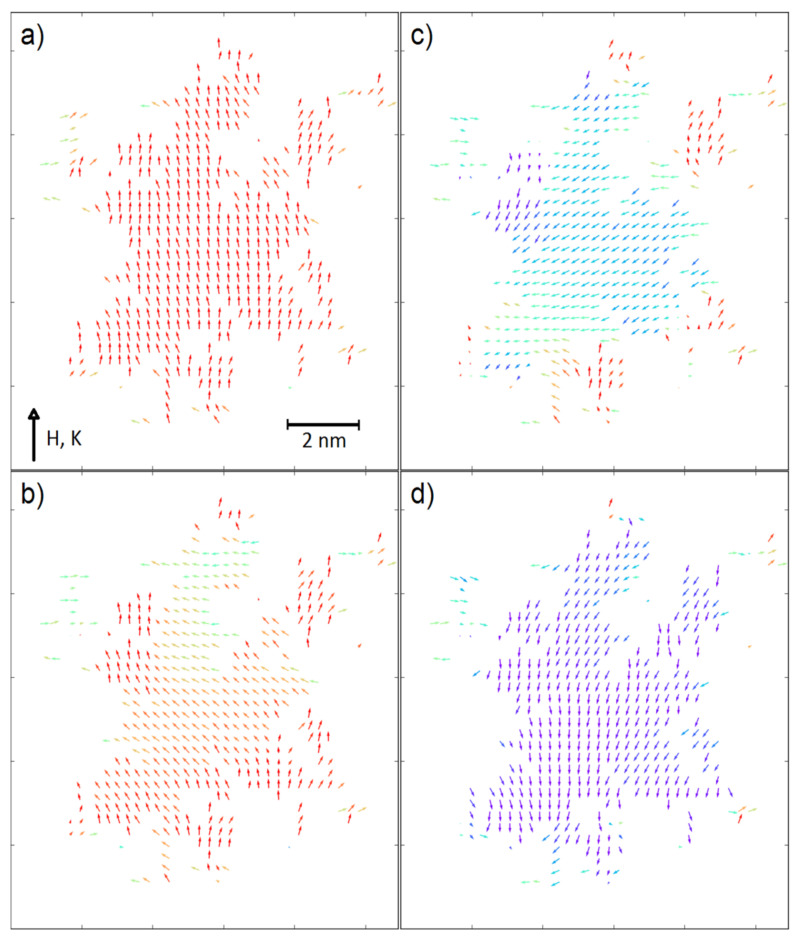
Spin configurations (cross-section) of the system presented in Figure 9 in the external magnetic field equal to (**a**) 1 T, (**b**) 0 T, (**c**) −0.4 T and (**d**) −0.6 T.

In order to complete this study, the simulated hysteresis loop as well as the spin configurations for the fractal exhibiting maximum of |*BH*|_max_ are presented in Figure 9 and Figure 10, respectively.

When the volume of the fractal is magnetically soft, the reverse magnetization process starts from the volume inside the system, similarly to the other soft magnetic fractal systems (see Figure 5). In these cases, the resulting non-zero anisotropy field appears as a competition between the volume magnetostatic energy and the magnetic anisotropy energy on the surface.

## 4. Concluding Remarks

Summarizing the presented simulations, one can state that the fractal like magnetic objects are very interesting regarding the possibility to optimize their hard magnetic properties. As it was shown, a combination of the fractal characteristics (i.e., size, surface development, magnetic parameters in volume and on surface) can lead to a change of coercivity mechanism. This effect might cause either improvement or deterioration of the fractal hard magnetic properties. In the case of ultra-high coercive objects, nanosized structure and fractal-like shape is not favorable because the surface magnetic moments make the reverse magnetization process more “easy”. When the anisotropy field of the volume is not extremely high, the change of coercivity mechanism may result in the observed increase of coercivity. Such a desired effect requires low dimensional fractals with a highly developed surface as well as relatively high surface magnetic anisotropy.

Quite interesting results have been obtained in the case of fractals with magnetically soft volume. Despite the fact that the presented analysis is only a rough estimation, one can define the system parameters that resulted in significant enhancement of hard magnetic properties, filling the gap between the conventional and rare-earth-based permanent magnets.

## Figures and Tables

**Table 1:** Values of the simulation parameters and scaling roles used in this work.

Parameter	Base Values	Scaling Rules
Scaling factor	from 1 to 100	-
Distance between nodes	0.28 nm	*r’ = r* × *n*
Dipolar constant	2.15 × 10^−7^ eVnm^3^	*D’ = D*
Anisotropy constant (surface)	from 0 eV to 5 × 10^−3^ eV	*K’_S_ = K_S_* × *n^2^*
Anisotropy constant (volume)	from 0 eV to 5 × 10^−4^ eV	*K’_V_ = K_V_* × *n^3^*
Spin	from 0.1 to 1	*S’ = S* × *n^3^*
Exchange integral parameter	1.5 × 10^−3^ eV	*J’ = J* × *n^−4^*

## Data Availability

The data presented in this study are available on request from the corresponding author.
